# Kidney dysfunction, systemic inflammation and mental well-being in elderly post-myocardial infarction patients

**DOI:** 10.1186/s40359-016-0170-z

**Published:** 2017-01-12

**Authors:** Rick H. M. Heeres, Ellen K. Hoogeveen, Johanna M. Geleijnse, Janette de Goede, Daan Kromhout, Erik J. Giltay

**Affiliations:** 1 Department of Psychiatry, Leiden University Medical Center, Postbus 9600, 2300 RC Leiden, Netherlands; 2 Departments of Internal Medicine and Nephrology, Jeroen Bosch Hospital, Den Bosch, Netherlands; 3 Division of Human Nutrition, Wageningen University, Wageningen, Netherlands

**Keywords:** Kidney dysfunction, Systemic inflammation, Apathy, Depressive symptoms, Dispositional optimism, Myocardial infarction

## Abstract

**Background:**

The aim was to investigate whether mild kidney dysfunction and low-grade inflammation in post-myocardial infarction patients are independently associated with markers of mental well-being (i.e. depressive and apathy symptoms, and dispositional optimism).

**Methods:**

In post-myocardial infarction patients, kidney function was assessed by estimated glomerular filtration rate (eGFR) calculated from the combined CKD-EPI formula based on serum levels of both creatinine and cystatine C. Systemic inflammation was assessed using high sensitivity C-reactive protein (hs-CRP) levels. The 15-item Geriatric Depression Scale (GDS-15), the 3-item apathy subscale and the 4-item optimism questionnaire (4Q) were used to measure mental well-being and were analyzed using linear multivariable regression analysis.

**Results:**

Of the 2355 patients, mean age was 72.3 (range 63–84) years and 80.1% were men. After multivariable adjustment, a poorer kidney function was associated with more depressive symptoms (β = -0.084, *p* < 0.001), more apathy symptoms (β = -0.101, *p* < 0.001), and less dispositional optimism (β = 0.072, *p* = 0.002). Moreover, higher levels of hs-CRP were associated with more depressive symptoms (β = 0.051, *p* = 0.013), more apathy symptoms (β = 0.083, *p* < 0.001) and less dispositional optimism (β = -0.047 *p* = 0.024). Apathy showed the strongest independent relation with both low eGFR and high hs-CRP.

**Conclusions:**

In post-myocardial infarction patients, impaired kidney function and systemic inflammation showed a stronger association with apathy than with depressive symptoms and dispositional optimism.

## Background

Depressive symptoms in coronary heart disease (CHD) patients are associated with an increase in cardiac morbidity and mortality [1]. The prevalence of major depressive disorder (MDD) in post-myocardial infarction patients is estimated at about 20% [2]. CHD is also associated with an increased occurrence of chronic kidney disease (CKD) [3], which may further contribute to the development of depressive symptoms [4–6]. It is well established that end-stage renal disease (ESRD) is associated with a poor quality of life and depressive symptoms, whereas this has not been thoroughly investigated for earlier stages of CKD. Patients with ESRD also have a prevalence of about 20% for MDD [7]. Moreover, as an inflammatory reaction is common in patients with ESRD, it is hypothesized that inflammation partly links ESRD to depressive symptoms [8, 9]. Systemic low-grade inflammation is present in patients with CKD, as well as in CHD, and may mediate the increased risk of depressive symptoms and apathy [10–13].

In three large cross-sectional studies, including 3700 to ≥ 28,000 patients [14–16], those with severe CKD (i.e. with an estimated glomerular filtration rate [eGFR] <30 ml/min/1.73 m^2^) had more depressive symptoms than those without CKD (eGFR ≥ 60 ml/min/1.73 m^2^). However, in only one of those studies (that included patients with diabetes) did the increased risk of depressive symptoms remain significant after adjustment for all relevant covariates [15]. Whether or not less severe forms of impaired kidney function (30 ml/min/1.73 m^2^ or higher) are associated with more depressive symptoms is still largely unknown. Moreover, the largest study that analyzed the continuous relation between eGFR and depressive symptoms found no significant associations [14]. Moreover, we are aware of only two studies on the association between CKD and depressive symptoms in cohorts of cardiac patients. In 374 patients suffering from congestive heart failure, Hedayati et al. found that those with severe CKD scored significantly higher on depressive symptoms [17]. In contrast, among 967 CHD patients, Odden et al. found no significant difference in depressive symptoms between patients with and without CKD [18].

A low-grade pro-inflammatory state is common in patients with CHD, exemplified by higher levels of serum C-reactive protein (CRP) [13]. A positive association between inflammatory markers and depressive symptoms is also well established in persons without somatic illness [19]. However, less is known about these associations in CHD patients. Among three studies that included from 71 to almost 1000 CHD patients [20–22], only the smaller study found a significantly increased CRP level in depressed CHD patients [22], whereas the two larger studies found no significant relation [20, 21]. In another study, when examining the linear relation between increasing CRP level and depressive symptoms, higher levels of CRP showed a significant association with more depressive symptoms; however, this association did not persist after adjustment for covariates [23].

The association between depressive symptoms and CHD has been called into question by recent cross-sectional and prospective studies in community-dwelling elderly [24–26]. Apathy, rather than depressive symptoms, was significantly associated with a higher incidence of CHD and other cardiovascular diseases. Apathy is a syndrome of primary motivational loss which manifests itself in a decrease in goal-directed cognition, emotion and behavior [27]. Two cross-sectional studies (including 1810 and 3534 subjects, respectively) also found that higher levels of CRP were significantly associated with more apathy [25, 26]. However, two longitudinal studies found no such association in 225 and 1015 elderly patients, respectively [28, 29].

The Alpha Omega Trial provided us with the opportunity to perform a cross-sectional analysis of mental well-being in post-myocardial infarction patients with a kidney function that ranged from normal to non-dialysis-dependent CKD stage 5. We aimed to investigate whether mild kidney dysfunction and low-grade inflammation were independently associated with markers of mental well-being i.e. depressive symptoms, apathy symptoms, and dispositional optimism. Dispositional optimism is defined as generalized positive outcome expectancies and a future orientation with trait-like mental properties [30].

Whereas previous studies used only creatinine levels to assess the eGFR, we included both creatinine and cystatin-C (CysC) levels to more accurately estimate kidney function [31], as well as high-sensitivity CRP (hs-CRP) as a measure of systemic inflammation.

## Methods

### Patients

The Alpha Omega Trial was a multicenter, double-blind, placebo-controlled trial conducted between 2002 and 2009 on the effect of low doses of n-3 fatty acids on cardiovascular events, described in detail elsewhere [32, 33]. The cohort consisted of 4837 free-living Dutch post-myocardial infarction patients aged 60–80 years, receiving state-of-the-art antihypertensive, antithrombotic and lipid-modifying drug treatment at baseline. Patients were randomly assigned to one of four trial margarines and were followed-up for 40 months (ClinicalTrials.gov no. NCT00127452). Blood samples were taken at baseline and after 40 months in patients randomized before August 2005 (i.e. 48% of the cohort, owing to financial constraints). Mental well-being questionnaires were administered at 40 months.

For the present cross-sectional study we used the 40-month follow-up data of 2355 (48.7% of 4837) patients who supplied blood samples and also completed the mental well-being questionnaires at 40 months [33].

### Assessment of psychological measures

Depressive symptoms were assessed using the 15-item Geriatric Depression Scale (GDS-15) [34]. This self-administered questionnaire contains 15 yes/no questions and excludes certain somatic symptoms which might be due to medical illness. Higher scores (range 0–15) indicate more depressive symptoms during the past week. The GDS-15 is a reliable and well-validated measure of depressive symptoms in elderly medical patients [35]. For computation of the GDS-15 score, two missing items were allowed and were subsequently imputed with the mean of the remaining 13 or 14 items (*n* = 206 of 2355, 8.7%).

Apathy was assessed using the GDS apathy-3 subscale. This is a subscale of the GDS-15 which consists of the following three questions: “Have you dropped many of your activities and interests?”, “Do you prefer to stay at home, rather than going out and doing new things?”, and “Do you feel full of energy?” Compared with the 14-item apathy scale [36], the GDS apathy-3 subscale has a sensitivity of 69% and a specificity of 85% [24]. For computation of the GDS apathy-3 subscale, one missing item was allowed and was subsequently imputed with the mean of the remaining two items (*n* = 73 of 2355, 3.1%).

Dispositional optimism was assessed using the 4Q which centers around a focus on the future and is more closely related to life engagement, vitality, and feelings of having a purpose in life. In the Netherlands, the 4Q was used as part of a national ‘life situation’ survey by the Central Bureau of Statistics in 1976 and 1982. The 4Q consists of the following four items “I still expect much from life,” “I do not look forward to the years to come,” “My days seem to pass by slowly,” and “I am still full of plans” (our translations). The response format was a 3-point scale of frequency: “fully in agreement” (score, 0), “partially in agreement” (score, 1), and “not in agreement” (score, 2). The additional answer category “do not know” was also coded as the midpoint (score, 1). The scores of two questions needed to be reverse coded, so that higher scores indicated greater optimism. Higher scores (range 0–8) indicated higher dispositional optimism. The 4Q shows moderate internal consistency and has clinical predictive value for cardiovascular mortality [37]. For computation of the 4Q score, one missing item was allowed and was subsequently imputed with the mean of the remaining 3 items (*n* = 21 of 2355, 0.9%).

### Assessment of biological measures

Blood handling procedures for the Alpha Omega Trial are described in detail elsewhere [38]. In brief, blood samples were obtained at the patient’s home or at the hospital, packaged in sealed envelopes and sent via standard postal service to a central laboratory.

Serum creatinine and CysC were measured from stored blood samples in a central laboratory [31]. Serum creatinine was measured by the modified kinetic Jaffé method (Dimension Vista 1500 Analyzer; Siemens). We calibrated directly to the standard supplied by the manufacturer from the National Institute of Standards and Technology Standard Reference Material, and a post-calibration correction factor was applied [31]. Intra- and inter-assay variations for low creatinine (mean = 0.8 mg/dl) were 1.8 and 2.9%, respectively, and for high creatinine (mean = 3.9 mg/dl) they were 0.8 and 2.2%, respectively. Serum creatinine values < 0.6 mg/dl were unreliable (owing to technical failure or analytical disturbance; *n* = 82) and, therefore, not reported in accordance with the Standard Operating Procedure of the central laboratory.

Serum CysC was measured by means of a particle-enhanced immune nephelometric assay (NLatex Cystatin C, Dimension Vista 1500 Analyzer; Siemens). We used calibrators and assays of the same lot code, which was stable (no downward drift). CysC was calibrated directly using the standard supplied by the manufacturer (traceable to the International Federation of Clinical Chemistry Working Group for Standardization of Serum Cystatin C) [39]. The analytical measurement range of CysC was 0.23–8.00 mg/L. Intra- and inter-assay variations for low CysC (mean = 1.00 mg/L) were 1.3 and 4.2%, respectively, and for high CysC (mean = 1.75 mg/L) they were 2.9 and 2.8%, respectively.

We estimated GFR using the combined creatinine-CysC-based Chronic Kidney Disease Epidemiology Collaboration (CKD-EPI) equation from 2012, taking into account age, sex, and race [31]. In addition, we used the CysC-based eGFR from the CKD-EPI equation from 2012 as well as the creatinine based eGFR from the CKD-EPI equation from 2009 separately [31].

Serum hs-CRP was measured in stored serum samples (Nephelometric, Deimension Vista 1500 analyzer, Siemens). Intra- and inter-assay variation for low hs-CRP (mean = 1.0 mg/l) was 2.1 and 1.6%, respectively, and for high hs-CRP (mean = 2.8 mg/l) this was 2.0 and 2.4%, respectively.

### Data collection

Patients were interviewed and physically examined by trained research nurses at home or in the hospital. Information on demographic variables (i.e., age, sex, ethnicity, educational level), lifestyle habits (i.e. smoking status, alcohol use, and physical activity), medication use and medical history (i.e., time since myocardial infarction, and history of stroke) were collected by self-administered questionnaires, as previously described in detail [33]. Medication was coded according to the Anatomical Therapeutic Chemical Classification System (ATC). Anthropometric measures were measured, body mass index (BMI) was computed (kg/m^2^) and blood pressure was measured. Diabetes mellitus was considered present in the case of a self-reported physician diagnosis, use of antidiabetic drugs, and/or elevated blood glucose.

### Data analysis

Patients were divided according to eGFR ≤ 60 or ≥ 60 ml/min per 1.73 m^2^ and also divided according to the hs-CRP level (<3 mg/l and ≥3 mg/l) based on the clinical cut-off point of 3 mg/l. Patient characteristics are listed in Table 1; data are presented as median (interquartile range), mean (±SD), or number (percentage of the total), where appropriate.

**Table 1:** Characteristics of the cohort of 2355 post-myocardial infarction patients according to creatinine-cystatin C-based eGFR and serum CRP level

	Creatinine-Cystatin C-based eGFR, ml/min/1.73 m^2^			C-reactive protein levels, mg/L		
	≥60 (*n* = 1750)	<60 (*n* = 605)	*P*-value	<3 (*n* = 1564)	≥3 (*n* = 791)	*P*-value
Age, years	71.2 ± 5.04	75.4 ± 5.3	<0.001	72.0 ± 5.3	72.7 ± 5.6	0.004
Men, No. (%)	1465 (83.7%)	422 (69.8%)	<0.001	1291 (82.5%)	596 (75.3%)	<0.001
Higher education ^a^, *n* (%)	767 (44.0%)	238 (39.7%)	0.064	715 (45.9%)	290 (36.9%)	<0.001
Body mass index^b^, kg/m^2^	27.6 ± 3.6	28.1 ± 4.1	0.003	27.3 ± 3.4	28.6 ± 4.15	<0.001
Physically active^c^, *n* (%)	389 (22.3%)	87 (14.5%)	<0.001	340 (21.8%)	136 (17.3%)	0.010
Current smoker, *n* (%)	267 (15.3%)	83 (13.8%)	0.363	184 (11.8%)	166 (21.0%)	<0.001
Alcohol use ≥1 glass/week, *n* (%)	1286 (73.6%)	360 (59.9%)	<0.001	1135 (72.8%)	510 (64.8%)	<0.001
Antidepressant use, *n* (%)	78 (4.5%)	40 (6.6%)	0.036	67 (4.3%)	51 (6.4%)	0.023
Time since myocardial infarction, years	4.5 ± 3.1	4.7 ± 3.4	0.110	4.5 ± 3.2	4.7 ± 3.2	0.117
Self-reported history of stroke, *n* (%)	94 (5.4%)	58 (9.7%)	<0.001	84 (5.4%)	68 (8.7%)	0.003
Diabetes^d^, *n* (%)	93.1 (22.3%)	188 (31.1%)	<0.001	375 (24.0%)	204 (25.8%)	0.334
Systolic blood pressure, mmHg	142.9 ± 20.5	141.6 ± 23.7	0.188	143.4 ± 20.9	140.9 ± 22.1	0.006
Use of cardiovascular medication^e^, *n* (%)						
Antithrombotic agents	1713 (97.9%)	584 (96.5%)	0.063	1529 (97.8%)	768 (97.1%)	0.322
Blood pressure lowering drugs	1729 (98.8%)	591 (97.7%)	0.051	1537 (98.3%)	783 (99.0%)	0.176
Statins	1553 (88.7%)	513 (84.8%)	0.011	1399 (89.5%)	667 (84.3%)	<0.001
High-sensitivity C-reactive protein, mg/L	1.42 (0.71-3.15)	3.33 (1.27-6.91)	<0.001	1.01 (0.61-1.72)	5.66 (4.04-8.83)	<0.001
Serum creatinine^f^, mg/dl	0.96 ± 0.18	1.54 ± 0.61	<0.001	1.05 ± 0.33	1.22 ± 0.56	<0.001
Serum cystatin C, mg/L	0.89 ± 0.13	1.41 ± 0.42	<0.001	0.96 ± 0.24	1.15 ± 0.42	<0.001

Data are presented as median (interquartile range), mean (± SD) or number (percentage of the total)

^a^Defined as higher vocational education, college or university

^b^Body mass index was calculated as weight in kilograms divided by height in meters squared

^c^Defined as ≥3 Metabolic Equivalent Tasks (MET) during >5 days/week

^d^Diabetes was considered to be present if a patient reported having received the diagnosis from a physician, was taking antidiabetic drugs, or had an elevated plasma glucose level (≥126 mg/dl in the case of patients who had fasted more than 4 h or ≥200 mg/dl in the case of non-fasting patients)

^e^Antithrombotic agents ATC code B01. Blood pressure lowering drugs: ATC codes C02, C03, C07, C08 and C09. Statins: ATC code C10AA

^f^To convert the values for creatinine to μmol/L, multiply by 88.40

Four eGFR categories were defined based on eGFR; ≥ 90, 60–89, 30–59, and < 30 ml/min/1.73 m^2^, using the CKD classification as proposed by the National Kidney Foundation-Kidney Disease Outcomes Quality Initiative (KDOQI) guidelines [40]. Clinical cut-points of CRP for risk of future cardiovascular events were used; levels <1, 1–3, 3–5, and ≥5 mg/L represent very low, low, mildly increased, and moderately to severely increased levels, respectively [41].

Because plasma concentrations of hs-CRP showed a positively skewed distribution, levels were log_e_ transformed before analyses to yield a normal distribution. Mean GDS-15 and 4Q scores ± standard error as well as median (25th and 75th percentiles [P_25_, P_75_]) were calculated per eGFR and hs-CRP category. The Pearson’s correlation coefficient was used to assess the association between the 12-item GDS depression score (excluding the 3 apathy items) and the 3-item GDS apathy score, as well as the association between the total GDS total score and the 4Q optimism score. The Pearson’s correlation coefficient was calculated between the original optimism score and an optimism score in which all items with a “don’t know” answer were imputed with the mean of the remaining items. A correlation coefficient of 0.95 (*P* < 0.001) was found in the 463 participants with at least one “don’t know” answer, suggesting that “don’t know” coded as the midpoint was unlikely to have distorted the total optimism score. Analysis of covariance (ANCOVA) was used to determine significance for linear trend over the four categories of eGFR and CRP levels. All adjusted analyses were corrected for the 4 randomized groups (because, in the original trial, patients were treated during 40 months with either n-3 fatty acids or placebo) [33].

Models were specified for multivariable testing to adjust for possible confounders. Model 1 adjusted for age, sex, level of education, and the 4 randomized treatment groups; model 2 additionally adjusted for BMI, smoking status, alcohol use, antidepressant use, time since myocardial infarction, stroke and diabetes; model 3 was the full model and additionally adjusted for the other possible predictor variables (either creatinine-CysC-based eGFR or hs-CRP level) to determine the independent association of eGFR and hs-CRP with depressive and apathy symptoms, and dispositional optimism. Moreover, we included all three mental well-being parameters (12-item GDS depression score (excluding the 3 apathy items), 3-item GDS apathy score and the 4Q optimism score) into one statistical model as independent variables with eGFR or hs-CRP as the dependent variables. Linear associations were tested using univariate regression analysis. Analyses were stratified for gender, and gender × predictor interaction terms were added to the full model 3 to test whether associations showed a significant difference between women and men.

Two additional sensitivity analyses were performed in which, first, we excluded all 221 participants with any missing items on any of the mental well-being questionnaires and, second, we also excluded all 463 participants with a “don’t know” answer on the 4Q as well as the 221 participants with any missing items, leaving 1723 participants available for the analyses.

All analyses were conducted using IBM SPSS statistics 20.0 (SPSS, Inc., Chicago, IL, USA).

## Results

In the total group of 2355 patients, mean age was 72.3 years; 80.1% were men and 99.8% were Caucasian. Of all patients, 1750 (74%) had an eGFR ≥ 60 ml/min/1.73 m^2^. The remaining 605 (26%) patients with an eGFR < 60 ml/min/1.73 m^2^ were more often female, less physically active, used less alcohol, more often had diabetes, and had a higher mean level of hs-CRP than those with an eGFR ≥ 60 ml/min/1.73 m^2^. There were 1564 (66%) patients with a low hs-CRP level of ≤ 3 mg/L; the 791 patients with a high hs-CRP level were more often female, more often smoked, and used less alcohol than those with low hs-CRP (Table 1).

The Pearson’s correlation coefficient showed that the association between the apathy score and the 12-item GDS depression score (excluding the 3 apathy items) was only 0.42. The complete 15-item GDS total score and the 4Q optimism score were inversely associated with a correlation coefficient of -0.63.

### Kidney function and mental well-being

In the fully adjusted model, categories with lower eGFR showed a significant association with more depressive symptoms. Patients with an eGFR < 60 ml/min/1.73 m^2^ had significantly more depressive symptoms (mean GDS-15 score 2.70) than patients with an eGFR ≥ 60 ml/min/1.73 m^2^ (mean GDS-15 score 1.80). Categories with lower eGFR showed a significant association with more apathy symptoms. Patients with an eGFR ≥ 90 ml/min/1.73 m^2^ showed significantly less apathy than patients with an eGFR of 60–89 ml/min/1.73 m^2^. Categories with lower eGFR were also significantly associated with less dispositional optimism (Table 2).

**Table 2:** Categories of creatinine-cystatin C-based eGFR and serum level CRP in relation to depressive and apathy symptoms, and dispositional optimism in 2355 post-myocardial infarction patients

Creatinine-cystatin C-based eGFR, ml/min/1.73 m^2^	≥90 (*n* = 517)	60 – 89 (*n* = 1233)	30 – 59 (*n* = 552)	<30 (*n* = 53)	Test statistic	*P*-value for trend
Depressive symptoms:						
Median (P_25_, P_75_)	1 (0 – 2)	1 (0 – 3)	2 (1 – 4)	2 (1 – 4)		
crude	1.54 (SE 0.08)^a^	1.92 (SE 0.06)^b^	2.68 (SE 0.12)^c^	3.12 (SE 0.41)^c^	F(1, 2351) = 80.6	<0.001
model 1	1.72 (SE 0.10)^a^	1.94 (SE 0.06)^a^	2.47 (SE 0.10)^b^	2.90 (SE 0.31)^b^	F(1, 2335) = 31.4	<0.001
model 2	1.79 (SE 0.10)^a^	1.95 (SE 0.06)^a^	2.34 (SE 0.10)^b^	2.67 (SE 0.31)^b^	F(1, 2280) = 17.4	<0.001
Apathy subscale:						
Median (P_25_, P_75_)	0 (0 – 1)	1 (0 – 2)	1 (0 – 2)	2 (1 – 2.5)		
crude	0.74 (SE 0.04)^a^	0.97 (SE 0.03)^b^	1.29 (SE 0.04)^c^	1.64 (SE 0.14)^d^	F(1, 2351) = 108.5	<0.001
model 1	0.85 (SE 0.04)^a^	0.98 (SE 0.03)^b^	1.18 (SE 0.04)^c^	1.47 (SE 0.14)^d^	F(1, 2335) = 36.1	<0.001
model 2	0.87 (SE 0.04)^a^	0.99 (SE 0.03)^b^	1.12 (SE 0.04)^c^	1.38 (SE 0.13)^c^	F(1, 2280) = 20.4	<0.001
Dispositional optimism:						
Median (P_25_, P_75_)	7 (6 – 8)	7 (6 – 8)	6 (5 – 7)	6 (4 – 7)		
crude	6.71 (SE 0.06)^a^	6.38 (SE 0.04)^b^	5.82 (SE 0.08)^c^	5.59 (SE 0.25)^c^	F(1, 2351) = 94.7	<0.001
model 1	6.49 (SE 0.07)^a^	6.36 (SE 0.04)^a^	6.04 (SE 0.07)^b^	5.91 (SE 0.22)^b^	F(1, 2335) = 21.6	<0.001
model 2	6.46 (SE 0.07)	6.35 (SE 0.04)	6.08 (SE 0.07)	6.01 (SE 0.22)	F(1, 2280) = 14.2	<0.001
C-reactive protein levels, mg/L	<1 (*n* = 766)	1 – 3 (*n* = 798)	3 – 5 (*n* = 330)	≥5 (*n* = 461)		*P*-value for trend
Depressive symptoms:						
Median (P_25_, P_75_)	1 (0 – 3)	1 (0 – 3)	1 (0 – 3)	2 (1 – 3.7)		
crude	1.86 (SE 0.08)^a^	1.87 (SE 0.07)^a^	2.15 (SE 0.13)^a^	2.55 (SE 0.12)^b^	F(1, 2351) = 28,34	<0.001
model 1	1.92 (SE 0.08)^a^	1.88 (SE 0.08)^a^	2.12 (SE 0.12)^a^	2.45 (SE 0.11)^b^	*F* (1, 2351) = 17.0	<0.001
model 2	1.97 (SE 0.08)	1.89 (SE 0.08)	2.07 (SE 0.12)	2.30 (SE 0.10)	*F*(1, 2280) = 6.7	0.010
Apathy subscale:						
Median (P_25_, P_75_)	1 (0 – 1,1)	1 (0 – 2)	1 (0 – 2)	1 (0 – 2)		
crude	0.88 (SE 0.03)^a^	0.98 (SE 0.04)^b^	1.05 (SE 0.05)^b^	1.26 (SE 0.05)^c^	F(1, 2351) = 42.8	<0.001
Model 1	0.90 (SE 0.04)^a^	0.99 (SE 0.03)^a^	1.04 (SE 0.05)^a^	1.21 (SE 0.05)^b^	*F* (1, 2335) = 27.6	<0.001
model 2	0.93 (SE 0.04)^a^	0.99 (SE 0.03)^a^	1.01 (SE 0.05)^a^	1.16 (SE 0.05)^b^	*F* (1, 2280) = 14.4	<0.001
Dispositional optimism:						
Median (P_25_, P_75_)	7 (6 – 8)	7 (6 – 8)	6 (5 – 8)	6 (5 – 7)		
crude	6.46 (SE 0.06)^a^	6.39 (SE 0.06)^a^	6.16 (SE 0.10)^b^	6.02 (SE 0.08)^b^	F(1, 2351) = 24.9	<0.001
model 1	6.40 (SE 0.06)	6.38 (SE 0.06)	6.17 (SE 0.09)	6.10 (SE 0.07)	*F* (1, 2335) = 12.8	<0.001
model 2	6.37 (SE 0.06)	6.38 (SE 0.06)	6.18 (SE 0.09)	6.16 (SE 0.07)	*F* (1, 2280) = 6.8	0.009

Data are reported as (adjusted) mean and standard errors (SE)

Analysis of covariance (ANCOVA) was used to determine significance for linear trend over the 4 categories of eGFR and CRP levels

Model 1: adjusted for age, sex, education, and 4 randomized groups (using 3 dummy variables)

Model 2: additionally adjusted for body mass index, smoking status, alcohol use, antidepressant use, statin use, time since myocardial infarction, stroke and diabetes

^abcd^ Superscript letters that are dissimilar indicate significant differences in post-hoc tests

Likewise, linear regression analyses showed that a lower level of eGFR was linearly associated with more depressive symptoms (β = -0.084, *p* < 0.001) and with less dispositional optimism (β = 0.072, *p* = 0.002; Table 3). The association between a lower level of eGFR and more apathy symptoms was stronger (β = -0.101; *p* < 0.001). All these associations persisted after additional adjustment for hsCRP. Interaction terms showed that relations were similar in women and men (*p* > 0.30, for all three interaction terms). When including all three mental well-being parameters into one statistical model as independent variables with eGFR as the dependent variable, apathy (β = -0.066, *p* = 0.001) was the only significant correlate, whereas dispositional optimism (*p* = 0.12) and the 12-item GDS depression score (*p* = 0.99) were no longer significant. Moreover, when we excluded the 48 participants with a eGFR < 30 ml/min/1.73 m^2^ from the analyses, the eGFR was still inversely associated with apathy (β = -0.054, *p* = 0.01), independently from CRP and all other covariates.

**Table 3:** Associations of creatinine-cystatin C-based eGFR and serum CRP level with depressive and apathy symptoms, and dispositional optimism in 2355 post-myocardial infarction patients

	Depressive symptoms			
	Crude	Model 1	Model 2	Model 3
Creatinine-cystatin C-based eGFR	-0.181	-0.116	-0.084	-0.074
	t = –8.904	t = -5.118	t = -3.691	t = -3.195
	*p* <0.001	*p* <0.001	*p* <0.001	*p* = 0.001
C-reactive protein levels	0.106	0.082	0.051	0.035
	t = 5.171	t = 4.017	t = 2.476	t = 1.650
	*p* <0.001	*p* <0.001	*p* = 0.013	*p* = 0.099
	Apathy subscale			
	Crude	Model 1	Model 2	Model 3
Creatinine-cystatin C-based eGFR	-0.219	-0.135	-0.101	-0.084
	t = -10.879	t = -6.002	t = -4.440	t = -3.598
	*p* <0.001	*p* <0.001	*p* <0.001	*p* <0.001
C-reactive protein levels	0.137	0.110	0.083	0.065
	t = 6.696	t = 5.479	t = 3.999	t = 3.039
	*p* <0.001	*p* <0.001	*p* <0.001	*p* = 0.002
	Dispositional optimism			
	Crude	Model 1	Model 2	Model 3
Creatinine-cystatin C-based eGFR	0.194	0.089	0.072	0.063
	t = 9.613	t = 3.979	t = 3.152	t = 2.693
	*p* <0.001	*p* <0.001	*p* = 0.002	*p* = 0.007
C-reactive protein levels	-0.094	-0.065	-0.047	-0.033
	t = -4.585	t = -3.244	t = -2.258	t = -1.556
	*p* <0.001	*p* = 0.001	*p* = 0.024	*p* = 0.12

Data are beta-coefficients (*p*-value) after linear regression analysis

CRP values were naturally log transformed before analysis because of a positively skewed distribution

Model 1: adjusted for age, sex, education, and 4 randomized groups (using 3 dummy variables)

Model 2: additionally adjusted for body mass index, smoking status, alcohol use, antidepressant use, statin use, time since myocardial infarction, stroke, and diabetes

Model 3: full model, additionally adjusted for the other possible predictors (Creatinine-cystatin C-based eGFR, or CRP levels)

### C-reactive protein and mental well-being

**Fig. 1:**
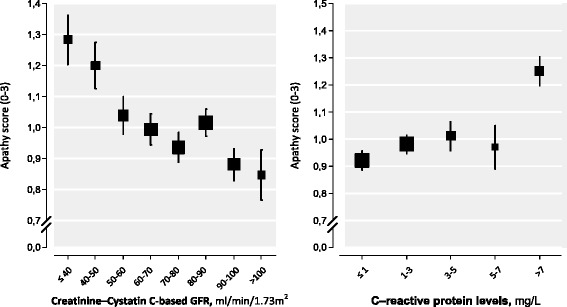
Associations of the creatinine-cystatin C-based eGFR and serum CRP levels with apathy in 2355 post-myocardial infarction patients. Mean serum eGFR and CRP levels are shown with error bars representing standard errors (SE), adjusted for age, sex, education, body mass index, smoking status, alcohol use, antidepressant use, statin use, time since myocardial infarction, stroke, and diabetes. The box sizes represent the relative number of patients

In the fully adjusted model, categories of higher hs-CRP serum level showed a significant association with more depressive symptoms, more apathy symptoms and less dispositional optimism (Table 2). Linear regression analysis also showed that a higher level of hs-CRP was significantly associated with more depressive symptoms (β = 0.051; *p* = 0.013) more apathy symptoms (β = 0.083; *p* < 0.001) and less dispositional optimism (β = -0.047; *p* = 0.024). However, after additional adjustment for the eGFR, only the association with apathy remained significant (β = -0.065; *p* = 0.002). In Fig. 1, the adjusted relationship with categories of hs-CRP seem to depict a threshold effect for the CRP level of ≥ 7. Moreover, when including all three mental well-being parameters into one statistical model as independent variables with the log-transformed CRP level as the dependent variable, apathy (β = 0.082, *p* < 0.001) was the only significant correlate, whereas dispositional optimism (*p* = 0.40) and the 12-item GDS depression score (*p* = 0.36) were not.

When testing for interaction, the interaction term for gender × hs-CRP was significant for depression (β = 0.129, *p* = 0.008), not significant for apathy (β = 0.076, *p* = 0.12), and of borderline significance for optimism (β = -0.088, *p* = 0.074). In subsequent stratified analyses, associations with depressive symptoms (β = 0.064, *p* = 0.007), apathy symptoms (β = 0.079, *p* = 0.001) and optimism (β = -0.059 *p* = 0.014) were stronger in men than in women.

Sensitivity analysis in 2134 and 1723 participants (i.e. excluding those with missing values and “don’t know” answer category) showed that the relationship between eGFR and CRP on the one hand, and depressive/apathy symptoms and optimism on the other, were hardly affected. In particular, there was no attenuation as, in the adjusted model, the relationship with CRP increased in strength in the sample of 1723 participants.

## Discussion

The present results show that kidney dysfunction (reflected by a lower eGFR) and systemic inflammation (reflected by a higher hs-CRP level) were independently associated with apathy in post-myocardial infarction patients. The associations with depressive symptoms and dispositional optimism were less robust. Kidney dysfunction showed a linear and somewhat stronger association for apathy than for systemic inflammation, for which there seemed to be a threshold value of 7 mg/L.

We are not aware of previous studies that assessed the relation between kidney dysfunction and apathy symptoms in a large cohort. Nevertheless, our findings are in line with others reporting that patients with an eGFR < 30 ml/min/1.73 m^2^ had more depressive symptoms than patients with an eGFR ≥ 90 ml/min/1.73 m^2^ [14–16]. We extended these findings by showing that an increase in depressive symptoms was already present in patients with an eGFR < 60 ml/min/1.73 m^2^ compared to those with a better eGFR. Low apathy was found within the normal range of kidney function. On the other hand, our results are in contrast to those of the only other large study on CHD patients (*n* = 967) that did not find more depressive symptoms in patients with an eGFR < 60 ml/min/1.73 m^2^ compared to those with an eGFR of ≥ 60 ml/min/1.73 m^2^ [18]. However, this latter study dichotomized kidney function at < 60 ml/min/1.73 m^2^ which reduced the statistical power, and did not include CysC levels or apathy in their analyses. A study in a general population sample of over 28,000 individuals showed an inverse, linear trend relation between kidney function and depression, which did not persist in the multivariable model [14]; however, in that study depression was measured using the 4-item Center for Epidemiologic Studies Depression Scale during a telephone interview and, again, neither CysC levels nor apathy were assessed. We have extended these findings by showing a linear trend relation between eGFR and depressive symptoms, which was strongest for the apathy subscale.

Several mechanisms could be involved in the relation between kidney dysfunction and apathy. For example, the vascular apathy hypothesis is suggested to underlie the relation between vascular disease and apathy symptoms [24]. Atherosclerosis may have caused renal vascular disease resulting in poorer eGFR as well as cerebrovascular disease causing brain ischemia which may result in apathy. The accumulation of uremic toxins causes encephalopathy in patients with kidney failure [42]. In patients with less severe kidney impairment, the neurotoxic effect of the accumulation of uremic toxins might have resulted in less extreme symptoms of apathy. Furthermore, anemia is a well-known complication of chronic kidney disease and could be part of the causal pathway to apathy. Also, apathy symptoms might overlap with other symptoms that occur in patients with kidney dysfunction. For example, fatigue shows some similarity with apathy and is one of the most frequently mentioned symptoms in patients with severe kidney dysfunction [43]. However, most studies on the relation between fatigue and psychological factors focused on depression and anxiety, but did not assess apathy. Finally, a third factor, related to aging, may underlie both apathy, inflammation and kidney dysfunction.

Earlier studies in CHD patients found no association between CRP levels and depressive symptoms [20, 23, 44]. However, the use of statins (and possibly beta-blockers and anti-depressants) may have reduced the inflammatory state [45–47] and thereby obscured the relation between CRP and depressive symptoms. In our study, however, an even higher proportion of patients used statins (87.7%) than in these latter studies; moreover, our study sample was larger than that of earlier studies [20, 23, 44]. Our findings are in line with the results of a large meta-analysis which also found that higher CRP levels were associated with more depressive symptoms in healthy individuals [19]. Also, for CRP levels we found that the strongest association was with the apathy subscale. Eurlings et al. found a concomitant relation between inflammation and apathy symptoms, and suggested that apathy symptoms may be part of sickness behavior, which can be described as a motivational state that reorganizes the organism’s priorities to cope with systemic inflammation and infectious disease [29]. Kidney dysfunction may have caused a higher systemic inflammatory state [8] which, in turn, resulted in more apathy. However, this hypothesis was only partly supported by our results which show that the relation between eGFR and apathy symptoms is largely independent of CRP levels.

As opposed to depressive symptoms, optimism has been associated with better health outcomes (such as survival) in CHD patients and the general population [48–51]. Both behavioral (e.g. healthy diet, physical activity, and non-smoking) and biological pathways have been suggested as mechanistic explanations. In CHD patients we found a significant relation between eGFR and dispositional optimism, and an inverse relation between CRP levels and dispositional optimism. Other studies found no relation between CRP and optimism or other measures of positive affect, but did find an inverse relation with other markers of inflammation, such as interleukin 6 (IL-6) and tumor necrosis factor alpha (TNF-α) [52–54].

Some limitations of the present study need to be addressed. First, because psychological measures were assessed at 40 months only, we were unable to address potential temporal relations. Second, because our cohort consisted of post-myocardial infarction patients, the results cannot be extrapolated to other populations. Third, depressive symptoms were assessed using a questionnaire with 15 relatively crude ‘yes/no’ answer categories. Fourth, because we lacked information on proteinuria or hematuria we could not distinguish between the presence or absence of chronic kidney disease in patients with an eGFR of 60–89 ml/min/1.73 m^2^.

However, our study also has several strengths. The study had a large enough sample size to detect small but significant effects. Moreover, we used the combined creatinine-CysC-based CKD-EPI equation to estimate the eGFR, which is considered more accurate than creatinine-based eGFR [31]. Also, use of the GDS-15 to assess depressive symptoms excluded certain somatic symptoms which might be due to medical illness in elderly medical patients [35]. Finally, it has been shown in cardiac patients that, compared with the Beck Depression Inventory (BDI) I and II, the GDS was better able to differentiate between those who are depressed and those who are not depressed [55].

## Conclusion

In conclusion, the present study shows a significant independent association between kidney dysfunction and apathy in post-myocardial infarction patients. This association was already apparent within the mild kidney dysfunction range. This implies that, at the population level, the impact of kidney dysfunction on mental well-being may be large, since we found that 26% of post-myocardial patients suffer from kidney dysfunction (eGFR <60 ml/min/1.73 m^2^).

## Abbreviations

4Q: 4-item Optimism Questionnaire
CHD: Coronary heart disease
CKD: Chronic kidney disease
CRP: C-reactive protein
CysC: Cystatin C
eGFR: Estimated glomerular filtration rate
ESRD: End stage renal disease
GDS-15: 15-item Geriatric Depression Scale
hs-CRP: High-sensitivity CRP
MDD: Major depressive disorder
